# 

**Published:** 1890-10

**Authors:** 


CONTENTS:

PAGE

ORIGINAL ARTICLES:

Some Recent Researches in the Diseases of the Domesticated Animals. By D. E. Salmon, D.V.M. . . . . 535

Veterinary Jurisprudence. By Prof. A. Liautard . . . . . 543

Contraction of the Horse's Foot. By Rush Shippen Huidekoper, M.D., Veterinarian . . . . . 553

Cotton-Seed Cakes. By J. C. Meyers, Sr. . . . . . 571

Moss as a Substitute for Linseed Meal and Hot Wooleu Cloths. By George H Berns, D.V.M. . . . . . 575

Age of the Horse, Ox, Dog, and other Domesticated Animals. By R. S. Huidekoper, M.D., Veterinarian . . . . . 578

SOCIETY PROCEEDINGS:

Pennsylvania State Veterinary Medical Association . . . . . 583

Keystone Veterinary Medical Association . . . . . 587

United States Veterinary Medical Association . . . . . 592

Editorial Note . . . . . 566

